# The influence of section diameter on the ultrasonic fatigue response of 316L stainless steel manufactured via laser powder bed fusion

**DOI:** 10.1038/s41598-025-97031-1

**Published:** 2025-04-18

**Authors:** Megan Trombley, Andrew Birnbaum, John Allison

**Affiliations:** 1 Department of Materials Science and Engineering, University of Michigan, Ann Arbor, MI 48109 USA; 2 Materials Science and Technology Division, United States Naval Research Laboratory, Washington, DC 20375 USA; 3 Department of Mechanical Engineering, University of Michigan, Ann Arbor, MI 48109 USA

**Keywords:** Size effects, High cycle fatigue (HCF), Residual stress, Surface condition, AISI 316 L, Probabilistic analysis, Metals and alloys, Mechanical properties

## Abstract

In this investigation, the influence of section diameter on high cycle fatigue (HCF) behavior of additively manufactured 316 L stainless steel was characterized. Three gauge-section diameters (5.0 mm, 2.5 mm, and 1.5 mm) were examined for their influence on the ultrasonic fatigue response of samples built via laser-powder bed fusion (L-PBF). HCF was conducted under full reversed loading (*R=−1*) conditions. A total of 130 specimens were characterized in the as-built state at maximum stresses ranging from 70 to 220 MPa. A Random Fatigue Limit (RFL) model using a Maximum Likelihood Estimation (MLE) was used to quantify statistical variability and estimate an S-N curve fit. The fatigue response shows that the largest gauge diameter (5.0 mm) resulted in the lowest fatigue strength at 89.5 ± 5.6 MPa, and the smallest diameter (1.5 mm) resulted in the highest fatigue strength at 122.0 ± 32.8 MPa. The 2.5 mm diameter specimens exhibited a fatigue strength of 98.7 ± 7.0 MPa. The primary failure mechanism in all as-built specimens was surface initiated cracking from crevices in the as-built surface finish. Additional specimens with a nominal diameter of 5.0 mm were fatigue tested with the as-built surface removed via low stress surface grinding. The fatigue strength of these samples increased to 170 MPa when 75 μm of the surface was removed and 179 MPa when the surface contour was entirely removed. Residual stresses were characterized by x-ray diffraction (XRD) and show a reduced axial residual stress with reduction in gauge diameter. Additional specimens were fatigue tested after undergoing a stress relief anneal, resulting in a 51% reduction in the residual stress and a 30% improvement in fatigue strength. An in-depth analysis of the microstructure, surface roughness, defects, and fracture surface indicate that both the surface condition and residual stress are the primary factors influencing the observed diameter effects on HCF.

## Introduction

Additive manufacturing (AM) is a rapidly evolving technology which allows for complex geometry, single-step manufacturing processes, and reduced assembly that offers many advantages over conventional manufacturing methods^1,2^. Laser-powder bed fusion (L-PBF) is one of the best-known metal AM methods, having been one of the first most widely used methods. In L-PBF, there are over 130 process variables that can contribute to the manufacture of a part^3,4^ each impacting the microstructure, mechanical behavior, and quality of the part. The establishment of quantitative understanding of these effects is important for development of integrated computational materials engineering (ICME) methods^5^ which have the potential to accelerate the design of robust AM processes and components^6,7^. In order to inform ICME models, the process-structure-property relationships for AM materials must be well understood. The most investigated process parameters are those which are easiest to control (e.g., laser power, scan speed, hatch spacing, layer thickness, laser spot size, scan strategy, etc.). Currently, most L-PBF equipment manufacturers designate ideal process parameters for a wide range of materials to reduce porosity, surface roughness, and manufacturing defects. These optimized parameters are based on research investigating the ways in which process parameters influence microstructures and properties^8–26^. For many structural components, fatigue is a critical design property and thus establishing quantitative understanding of the fatigue behavior of AM components is essential.

Fatigue of 316 L stainless steel in L-PBF has been widely researched^27–41^ with three main attributes being known to affect the fatigue behavior: as-built surface finish, residual stresses, and processing defects. In multiple studies on fatigue of AM samples, both surface roughness^2,33,35,42,43^ and internal defects^27,39^ were found to be significantly influential on fatigue behavior because they served as points of stress concentration^44,45^. The site for crack initiation is dictated by the stress-concentration factor, K_t_, whose value depends on the geometry of the defect^46^. Local cyclic stresses are also affected by the local residual stresses. In general, surface residual stresses increase the fatigue strength when they are compressive and decrease when tensile, particularly in hard steels^46^. Investigations into the residual stress states of L-PBF printed parts have shown axial tensile residual stresses reaching or exceeding the bulk room temperature yield stress of the wrought material^47^. Residual stresses arise from melting, solidification, and re-melting during the laser processing which leads to large thermal stress gradients^48–52^. Irrespective of processing parameters and orientation, in nearly every instance, axial residual stresses in L-PBF application of 316 L are generally compressive at the center of a sample and tensile at the surface^47,51–54^. This distribution of residual stresses can lead to part distortion and degradation of fatigue performance^41,49,55–57^.

One of the advantages of L-PBF AM is the ability to fabricate components with complex geometries which may have substantial variations in section thicknesses throughout the component. The influence of AM section thickness on fatigue behavior has not been the subject of significant study. In conventionally manufactured components, it has been shown that the fatigue strength of metallic materials can decrease with increasing specimen size^58–64^. The prevailing theory behind this phenomenon comes from Weibull’s weakest link theory, which postulates a larger volume will have a higher probability of shorter life due to the abundance of more crack initiating elements^44,63,65^. This phenomenon has not been explicitly shown to occur in L-PBF components, although some aspects of this have been investigated. Studies have shown the effect of powder layer thickness^13,20,28,51^ and overall part height (number of layers)^51,53^ on structural and mechanical results in AM materials, but only limited investigation has been conducted on geometric scaling. In this instance, geometric scaling can be thought of as scaling in three dimensions such that similar geometries are maintained. The current investigation aims to characterize gauge section diameter effects occurring in 316 L L-PBF, as well as evaluate various aspects of processing-structure-property relationships to determine the degree of influence each has on these scaling effects.

Ultrasonic fatigue (UF) testing is used in this research for its ability to rapidly obtain high cycle fatigue (HCF, 10^4^ to 10^7^ cycles)^29,66–68^ and very high cycle fatigue (VHCF, >10^7^ cycles)^3,4,68–70^ data. UF testing is conducted by stimulating specimens at resonant frequencies close to 20 kHz^71,72^, compared to the conventional servo-hydraulic HCF testing apparatus which is typically conducted at 20–60 Hz. This reduces testing time in the HCF and VHCF regime to hours or days rather than months or, in the case of VHCF, years. This enables testing of a significantly larger number of samples which, in turn, substantially improves the statistical significance of inferences which can be made on the factors influencing fatigue responses. Prior research suggests that the frequency effects on the VHCF fatigue behavior in austenitic stainless steels are minimal^73^.

In this study, the effects of sample gauge section diameter on the ultrasonic fatigue behavior of 316 L SS produced via L-PBF are investigated. In particular, the HCF fatigue behavior was evaluated for three different gauge diameters (1.5 mm, 2.5 mm, and 5.0 mm) in L-PBF samples manufactured using identical processing parameters. These samples were also evaluated for changes in surface roughness, defect morphology, and microstructure that may arise due to changes in gauge diameter. The influence of surface roughness was examined by comparing as-built samples to samples with the surface removed by low stress grinding. As-built residual stress distributions were also characterized for two different gauge diameters (1.5 mm and 5.0 mm). In the 5.0 mm gauge diameter sample the influence of stress relieving heat treatment on HCF and residual stress was quantified.

## Materials and methods

### Material properties

A single batch of AISI 316 L stainless steel powder particles produced by GE Additive Concept Laser GmbH (CL 20ES) was used to fabricate all specimens. The powder was reported by GE Additive to have a chemical composition shown in Table 1 which is generally consistent with standard AISI 316 L stainless steel used for L-PBF^74^. A combination of virgin and sieved particles were used in the fabrication of all specimens. Particle size analysis revealed the median particle size to be approximately 30 μm with 90% of particles being less than 45 μm (Table 2). Additionally, 73.6% of particles measured were found to have a sphericity of 0.9 or less, with the average sphericity being 0.791. A portion of the powder batch was used to fabricate L-PBF tensile samples to measure bulk mechanical properties. The results indicated that the elastic modulus for this condition was 165 GPa with a yield strength of 466 MPa, showing a significantly lower elastic modulus and higher yield strength compared to wrought 316 L stainless steel^75,76^(Table 3).

**Table 1:** Chemical composition (in wt%) of CL 20ES 316 L stainless steel powder particles according to GE additive compared to the ASTM standard composition for 316 L laser-powder bed fusion powder particles. Single values represent maximum allowable contents for that given element.

Type	Fe	Cr	Ni	Mo	C	Mn	*P*	S	Si
GE Additive	Balance	16.5–18.5	10.0–13.0	2.0-2.5	0.030	2.0	0.045	0.030	1.0

**Table 2:** Powder particle size (in µm) cumulative distribution function of GE additive CL 20ES stainless steel powder.

	10%	50%	90%
Virgin	21.5	30.4	43.5
Virgin + Sieved	20.2	30.1	44.8

**Table 3:** Mechanical properties of 316 L austenitic stainless steel at room temperature.

Type	Elastic modulus [GPa]	Tensile strength [MPa]	Yield strength 0.2% offset [MPa]
Wrought	193	558–560	290
Experimental	165	565	466

### Specimen fabrication

All fatigue specimens were fabricated using a GE Additive Concept Laser M2 (CL M2) at the US Naval Research Laboratory. The processing parameters, listed in Table 4, are the recommended parameters for 316 L by GE Additive.

**Table 4:** Laser powder bed fusion (L-PBF) process parameters for every build.

Machine	Laser power	Scan speed	Layer thickness	Laser spot size
CL M2	370 W	900 mm/s	25 μm	160 μm

**Fig. 1:**
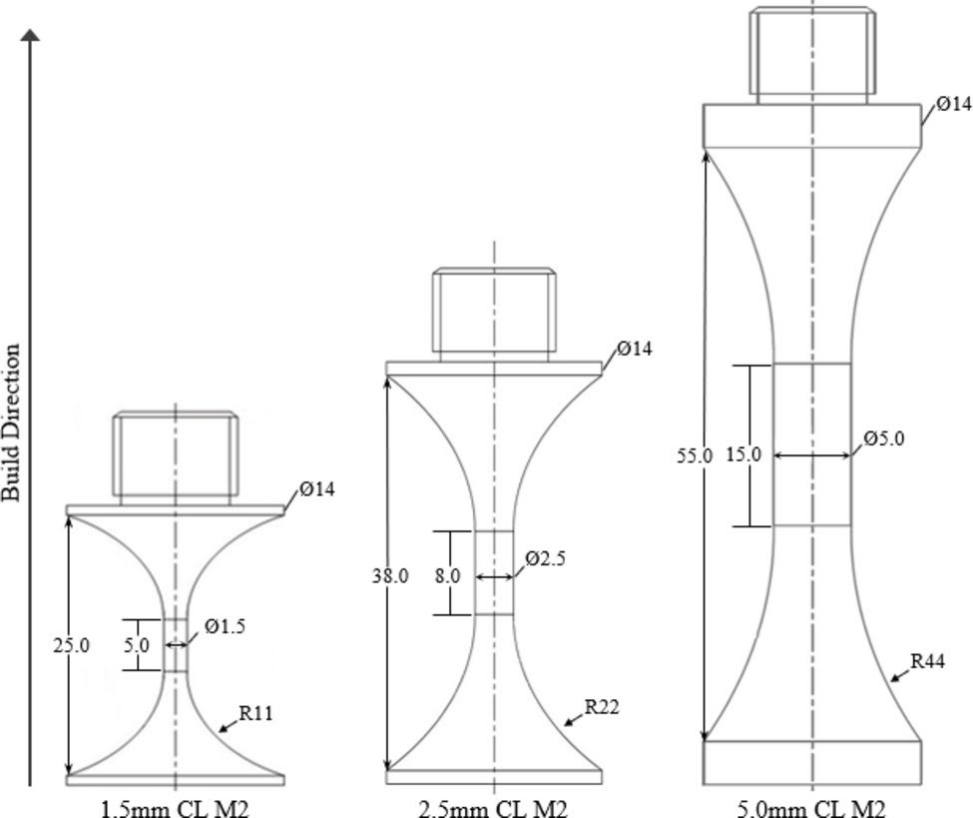
Specimen geometry for ultrasonic fatigue tests.

The specimens were fabricated in a cylindrical dog-bone geometry (Fig. 1) in the vertical orientation for ultrasonic fatigue testing. To test size effects, the gauge diameter was built to be 5.0 mm, 2.5 mm, or 1.5 mm, with the length of the specimen adjusted to maintain a 20 kHz resonant frequency for each gauge diameter. The diameters of these specimens were chosen to be small enough to mitigate the damping effects seen in VHCF of austenitic stainless steels^71^. L-PBF samples were fabricated in 4 different builds, with 32 to 40 samples per build. For each layer, individual fatigue samples were fabricated first by building the sample interior (infill region) with multiple line scans, followed by a number of final contour scans to fabricate the sample as-built surface. Individual samples were removed from the base using electro-discharge machining (EDM) prior to thread machining.

### Ultrasonic fatigue testing

Ultrasonic fatigue (UF) testing is conducted at room temperature on equipment developed by University of Natural Resources and Life Sciences, Vienna (BOKU)^72^ and operated at 20 kHz. Experimentation was conducted under fully reversed (R=−1) loading. All testing was done with forced-air cooling and pulse-pause loading, pulsing for 200 ms every 3000–5000 ms, in order to reduce specimen heating. Failure was defined as the point in life at which the UF instrumentation detects a change in frequency greater than 200 Hz from the starting resonant frequency of approximately 20 kHz. A value of 200 Hz was chosen to allow the crack to propagate sufficiently to be observed by the unaided eye but not fully fracture the specimen. If a specimen does not meet this failure criteria prior to 10^8^ cycles, it was deemed a runout.

The HCF testing protocol consists of four steps: (1) statistical sample of intermediate-stress level fatigue (~ 20 samples); (2) statistical sample of high-stress level fatigue (~ 10 samples); (3) quantification of fatigue strength at 10^8^ cycles using staircase testing at low-stress fatigue (~ 10 samples); and (4) application of a Random Fatigue Limit (RFL) model using a Maximum Likelihood Estimation (MLE) to quantify statistical variability and estimate the S-N curve and fatigue strength, S_N_.

### Fatigue strength calculations

A life-regression model (S-N curve) is used to quantitatively describe the fatigue properties of a given sample group from experimental fatigue tests. There are multiple models that can be used to generate an S-N curve from fatigue data, including the Random Fatigue Limit (RFL) model^77^ which is used herein. The RFL curve fit also acts as a metric to more readily determine the fatigue strength, S_N_, of a material condition by using the entire S-N curve population including runout data points. The RFL model can be used for a range of different distributions and constraints, making it important to implement a Maximum Likelihood Estimation (MLE) to the calculation of the RFL model. In the current investigation, this was done following the methods outlined by Engler-Pinto Jr. et al.^68^. MLE analysis has shown that a Weibull distribution is the best fit in most cases, so, in the current investigation this is the distribution used in each RFL analysis. Once the appropriate RFL model is selected, the fatigue limit and fatigue strength can be calculated for the dataset. The RFL model was selected in place of other models such as the Modified Basquin model^67,78^ as it has been shown to generally provide a better fit to HCF data as determined by MLE^67^.

Another common method for estimating the fatigue strength, S_N_, is the staircase method. This is a fatigue testing method which sequentially tests samples at varying stress levels. The first sample is tested at a pre-determined stress and observed to be either a failure or a runout. The following sample is tested at a higher stress if the previous sample was a runout and a lower stress if it was a failure. This continues for any number of samples, resulting in a roughly even split of runouts and failures. The median stress of these tests is used as an estimate of the median fatigue strength of the material, with the assumption that the fatigue strength is normally distributed. For its simplicity, this method was used to test a portion of the sample group, however, an RFL model is still applied to the entire dataset as it is more accurate in predicting the fatigue strength of data that is not normally distributed^67^.

### Surface roughness

Surface roughness was measured on all samples using a VHX-7000 Keyence optical microscope Ver 1.4.25.19. Both line roughness and surface roughness were measured from the included Keyence software (VHX Control System Ver 18.12.04.0 A Ver 01.00.00.04) on as-built dog-bone samples. A shape correction is applied to account for the cylindrical surface. Nine images of the surface were taken at 600x magnification and stitched together to form an area of interest approximately 900 × 1200 μm. Surface characteristics measured include the arithmetic mean height of the surface profile (S_a_), root mean square height of the surface profile (S_q_), and the maximum height of the surface profile (S_z_), as defined by ISO 25178-2.

### As-built surface removal

**Fig. 2:**
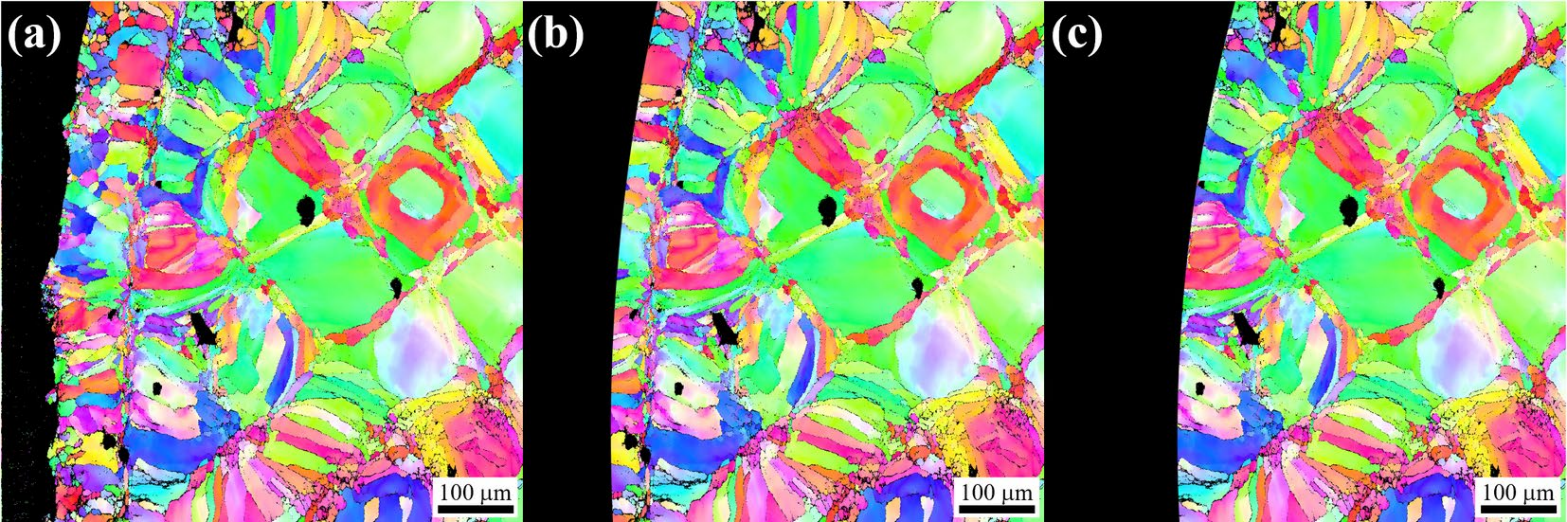
EBSD images showing cross sections of the as-built sample microstructure (**a**) and depictions of sample cross sections with surface removal (**b**), and with contour removal (**c**). In (a) the entire contour zone is intact, (**b**) depicts the removal of approximately 75 μm with the contour zone partially intact, and (**c**) the entire contour zone is depicted as removed.

Removal of the as-built sample surface was conducted to characterize both the impact of reducing the as-built surface roughness and also removing the L-PBF contour passes from the printed part. In both cases, material removal is done using a RTS Leeds low-stress sample polishing machine at Element Materials Technology in Wixom, MI. The CL M2 samples show a clear distinction between the infill and contour regions, with differing microstructures and defect concentrations, as shown in Fig. 2a. Removing the as-built surface was characterized into two groups: surface removal (Fig. 2b) and contour removal (Fig. 2c). The surface removal machines approximately 75 μm from the surface while the contour removal removes the entire contour passes (approximately 150 μm) from the surface.

### Residual stress

The axial residual stress was measured on three samples: one as-built 5.0 mm CL M2, one as-built 1.5 mm CL M2, and one stress relief annealed 5.0 mm CL M2. Residual stress measurements are done using x-ray diffraction (XRD) with material removal via electropolishing to get a profile of residual stress versus depth from the surface. Samples were measured using an LXRD 13,115 with a Mn target, x-ray elastic constant of 20,199 ksi (139,000 MPa), {311} crystallographic plane, and 152.8° Bragg angle. Residual stress measurements were conducted by Proto Manufacturing Inc. in Taylor, MI.

In addition to measuring the residual stress, a partial relief of the residual stress was conducted by heat treating eight 5.0 mm CL M2 samples. A stress relief anneal was completed in a Lindberg 1700°C tube furnace in a sealed Argon environment. The anneal consisted of a forty-five minute heat-up, four hour soak at 650°C, and a three hour furnace cool to room temperature. These conditions were chosen to provide stress relief while limiting microstructural changes^30,33,79–82^.

### Microstructure and fractography

Microstructural characterization was conducted to evaluate the influence of gauge diameter. The samples were cut using a low speed saw, ground using increasingly fine grit SiC grinding paper, and polished using 1 μm diamond suspension followed by 0.04 μm colloidal silica, using the procedure outlined by Rowenhorst et al.^83^. Sections were taken from the gauge area both parallel and normal to the build direction. Electron backscatter diffraction (EBSD) was used to evaluate the microstructure in multiple orientations. EBSD scans were taken in both the interior and at the edge of each sample to obtain an understanding of how the microstructure changes throughout the samples, most notably from the contour to the infill. EBSD characterization was accomplished using an EDAX Hikari EBSD camera on a Tescan MIRA-3 GMH electron microscope at 30 kV and a beam intensity of 18, with a scan area 600 X 600 μm and a step size of 0.5 μm. Analysis was completed using EDAX OIM Analysis version 8.6.0101 × 64 in the austenite phase with a minimum grain boundary misorientation angle of 1^o^.

Analysis of the fracture surface was conducted utilizing SEM on a Tescan Mira-3 GMH electron microscope. 3D analysis of the specimens and their fractures surfaces were done using a VHX-7000 Keyence optical microscope and its accompanying software. Analysis of defects on the fracture surface was completed with the use of SEM images and ImageJ.

## Results

### Microstructure

**Fig. 3:**
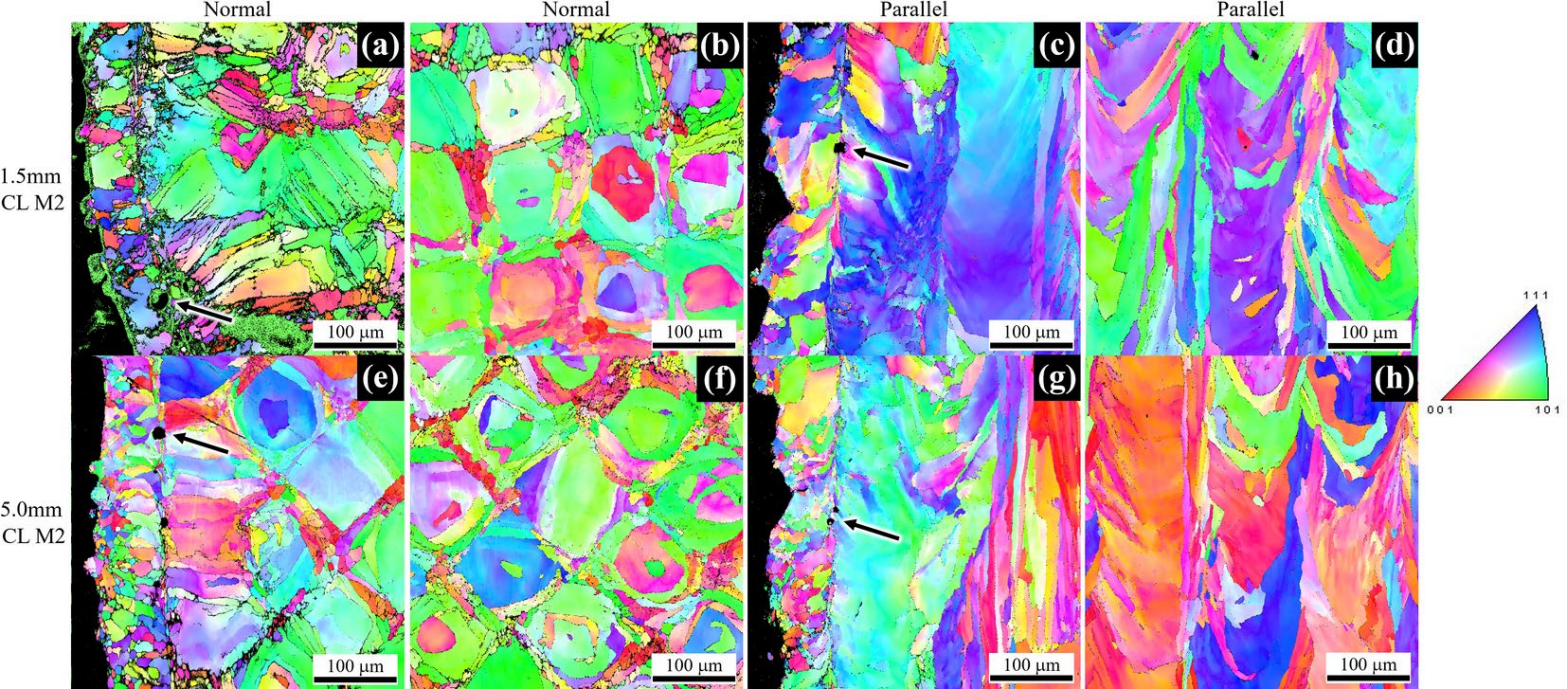
Inverse pole figure (IPF) maps generated via EBSD indicating the microstructure present in the (**a**-**d**) 1.5 mm CL M2 and (**e**-**h**) 5.0 mm CL M2 sample groups. Arrows indicate pore-like defects at the intersection of the infill and contour regions.

The microstructure was characterized for each unique build and a representative sample of images are shown in Fig. 3. The microstructure was evaluated both normal and parallel to the build direction to observe the anisotropic morphology. Each orientation was evaluated in the specimen interior and the near surface region (edge). This allowed for understanding of microstructural changes that may occur between the infill and contour regions. Figure 3a-d depicts the microstructure observed for the 1.5 mm CL M2 specimens for direct comparison to the 5.0 mm CL M2 specimens below (Fig. 3e-h). All samples show a distinct change in microstructure from the contour to the infill regions. In general, a uniform crosshatch patterning normal to the build direction and columnar grains parallel to the build direction in the infill region are seen. The contour region consists of generally smaller and more equiaxed grains. Additionally, an increase in the density of pore-like defects at the intersection of the contour and infill regions is observed as indicated by the arrows in Fig. 3a, c, e, and g.

### Surface roughness

**Fig. 4:**
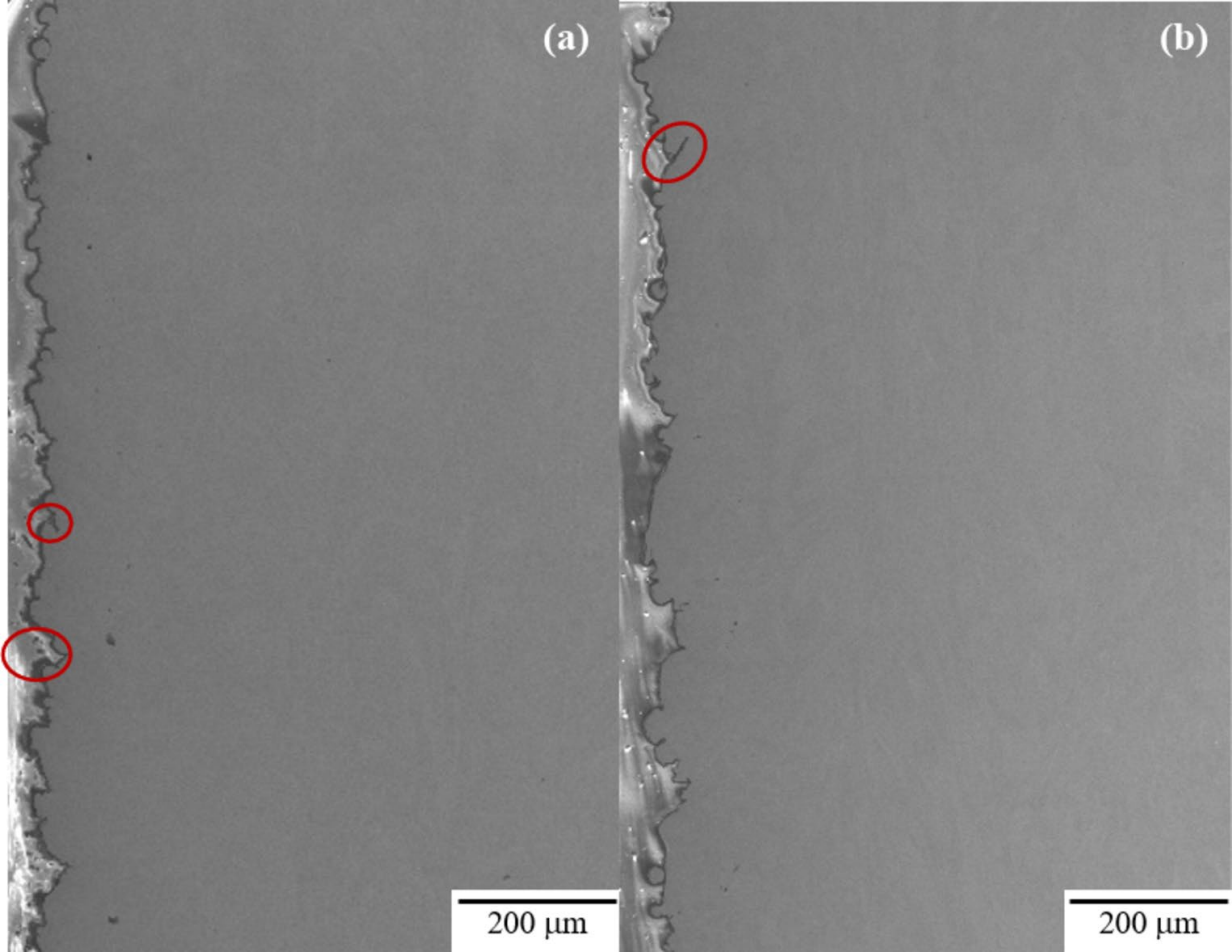
SEM profile view, perpendicular to the build direction for the as-built surface roughness in (**a**) 2.5 mm CL M2 and (**b**) 5.0 mm CL M2 samples. The red circles indicate the presence of surface crevices that are deeper than the surface roughness measurements shown in Table 5.

Surface roughness measurements indicated that there was no significant change in surface roughness as the diameter of the as-built sample is changed (Table 5). The as-built surface was also evaluated by examining cross-sectioned samples perpendicular to the build direction (Fig. 4) using SEM. The Keyence, with its limited resolution, measures surface roughness predominantly from the evidence of build layers and partially melted particles adhered to the sample surface. Using the SEM, evidence of individual particles and build layers, as well as deeper crevices (denoted in Fig. 4 with red circles) that are generally not picked up by the Keyence are seen. For this reason, future discussion will distinguish between the surface roughness (Keyence) and surface crevices (SEM).

**Table 5:** Surface roughness characteristics for each gauge diameter sample group. Characteristics include the arithmetic mean deviation of the surface profile, root mean squared deviation of the surface profile, and maximum height of the surface profile as defined by ISO 25178-2. At least 3 samples were characterized for each gauge diameter.

Type	S_a_ [µm]	S_q_ [µm]	S_z_ [µm]
1.5 mm CL M2	3.38 *± 0.87*	4.28 *± 1.05*	32.83 *± 5.73*
2.5 mm CL M2	3.11 *± 0.97*	3.92 *± 1.19*	29.35 *± 5.83*
5.0 mm CL M2	3.93 *± 0.49*	5.00 *± 0.67*	37.05 *± 5.78*

### Residual stress

**Fig. 5:**
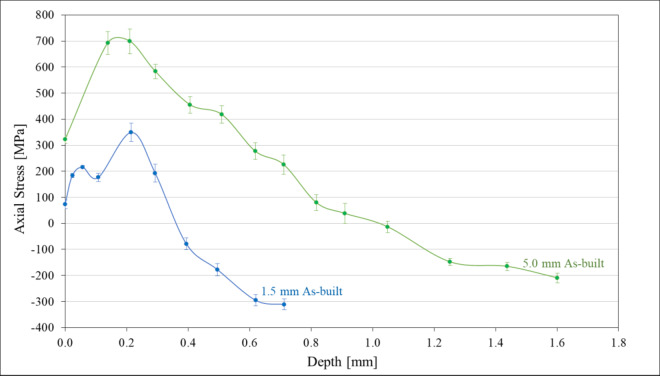
Comparison of the axial residual stress depth profile of a 1.5 mm and 5.0 mm as-built sample.

Axial residual stress measurements were taken on both a 1.5 mm and 5.0 mm sample. Measurements were made through the depth of the as-built samples, showing the residual stress profile throughout the thickness of the samples (Fig. 5). This shows that both samples have tensile residual stresses on the surface and compressive residual stresses in the sample interior. The magnitude of tensile residual stresses (at the sample edge) was significantly higher in the 5.0 mm sample and the magnitude of the compressive residual stresses (in the sample interior) was higher in the 1.5 mm sample. It should be noted that the residual stress profile in the 5.0 mm sample was measured prior to fatigue testing while the residual stress profile in the 1.5 mm sample was measured after the sample had been tested in fatigue and the sample fractured. This was required due to lack of additional unfatigued samples for the 1.5 mm sample diameter. In the 1.5 mm sample, residual stress measurements were taken well below the fracture surface while still being within the gauge length in order to avoid large deviations in residual stress due to stress relaxation that occurs during fatigue fracture. Axial tensile residual stress peaks just below the surface, exceeding the measured values of yield strength and ultimate tensile strength in the 5.0 mm sample. Despite this, no evidence of deformation or fracture was seen in these samples prior to testing. As suggested by Wu et al.^47^, comparisons between residual stress and the uniaxial yield strength may not be appropriate in AM materials due to the multiaxial nature of the residual stress states.

**Fig. 6:**
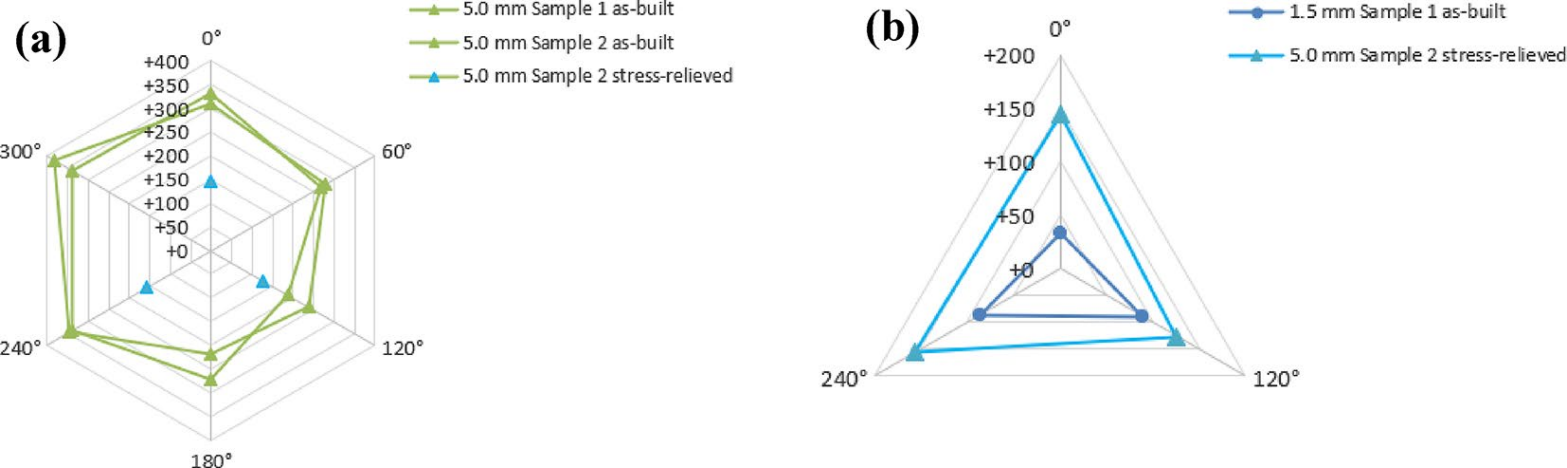
(**a**) Comparison of surface axial residual stress of 5.0 mm as-built and stress-relieve samples. (**b**) Comparison of 1.5 mm as-built and 5.0 mm stress-relieved samples.

Axial residual stress measurements are also taken on the surface of two 5.0 mm samples and one 1.5 mm sample. These measurements were made in either three or six different regions around the sample circumference. The surface axial residual stress measurements are non-destructive which allows for one of the samples to be measured before and after stress relief annealing. Figure 6a shows a comparison of the 5.0 mm samples measured in the as-built condition and stress relieved condition. Figure 6b shows how the surface axial residual stress of the 5.0 mm stress-relieved samples compares to the as-built 1.5 mm sample. Table 6 shows the stress-relieved 5.0 mm had lower magnitude tensile residual stress compared to the as-built 5.0 mm, but still a higher magnitude tensile residual stress compared to the as-built 1.5 mm. Due to lack of sample availability, residual stress measurements in the 2.5 mm CL M2 samples were not conducted.

**Table 6:** Averaged values of the measured surface axial residual stress for each sample type.

Type	Average measured stress [MPa]	Number of measurements
5.0 mm As-built	292 *± 13*	12
5.0 mm Stress-relieved	143 *± 11*	3
1.5 mm As-built	69 *± 17*	3

### Ultrasonic fatigue behavior

**Fig. 7:**
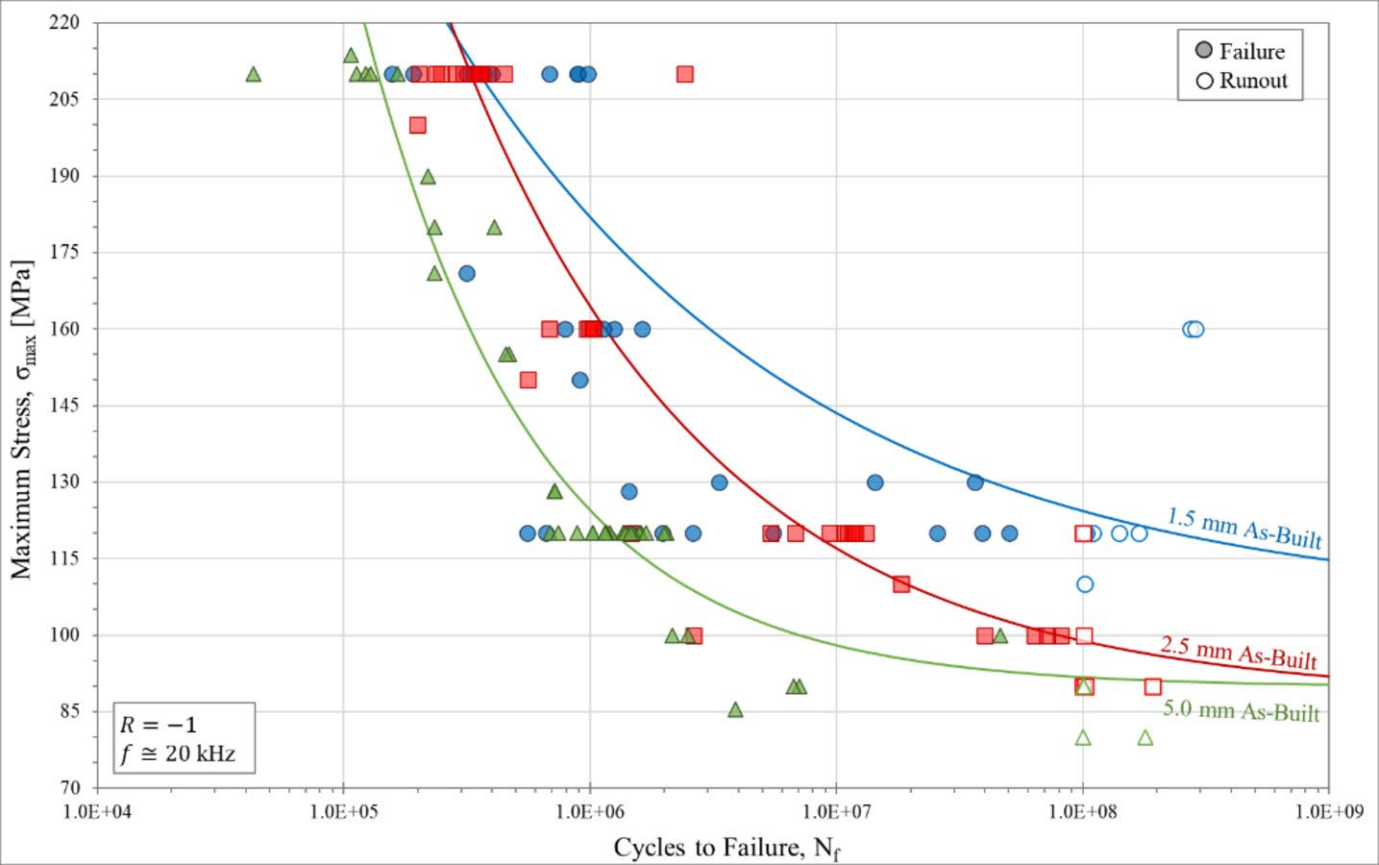
Stress-life (S-N) ultrasonic high cycle fatigue curves for all as-built CL M2 samples. Runout samples are indicated by unfilled data icons and are classified as cycling longer than 10^8^ cycles without failure. Each set of data is accompanied by a Weibull distribution curve fit determined via random fatigue limit (RFL) analysis.

A total of 130 as-built CL M2 specimens were tested for the three different gauge diameters (1.5 mm, 2.5 mm, and 5.0 mm). An additional eight stress-relief annealed, eight surface-removed, and eight contour-removed specimens were fatigue tested to show the influence of residual stress, surface roughness, and contour/infill defect density on fatigue behavior, respectively. Each group was UF tested across a range of maximum stress levels to capture behavioral changes at different stresses and to obtain a more complete view of the RFL estimated S-N curve. The UF results for each as-built CL M2 sample group are graphically represented in Fig. 7. Fitting each sample group to an RFL model assuming a Weibull distribution informed by MLE indicates that reasonable curve fits were obtained for all of the fatigue data groups. As shown in Fig. 7, significant improvements in fatigue behavior are observed with decreasing gauge diameter. From the RFL model curve fit, a value for the fatigue strength can be calculated along with a standard deviation of the data. The fatigue strength can also be calculated from the staircase testing procedure. Comparing the two methods of calculating the fatigue strength (Table 7) shows they are in good agreement. The fatigue results of Fig. 7 show that with decreasing gauge diameter, a general increase in fatigue life, N_f_, and fatigue strength, S_N_, at 10^8^ cycles, was observed. The variability for the 1.5 mm as-built samples is rather substantial, with a standard deviation for the calculated fatigue strength of 26.1 MPa (Table 7). The standard deviation in the RFL calculation is high in this case due to the two samples that were runouts at 160 MPa. Despite this variability, there is a statistically significant gauge diameter effect on fatigue behavior occurring between 5.0 mm and 2.5 mm samples (*p* < 0.0001) and between 2.5 mm and 1.5 mm samples (*p* = 0.0467).

**Table 7:** Fatigue strength for each sample group as determined by random fatigue limit (RFL) model and the staircase testing procedure. The fatigue strength is defined as the stress needed to cause failure at 10^8^ cycles.

	Fatigue strength [MPa] via RFL	Fatigue strength [MPa] via staircase
1.5 mm As-built	123.0 *± 26.1*	122.5 *± 6.6*
2.5 mm As-built	98.7 *± 7.0*	97.0 *± 6.4*
5.0 mm As-built	89.5 *± 5.7*	91.0 *± 7.0*

**Fig. 8:**
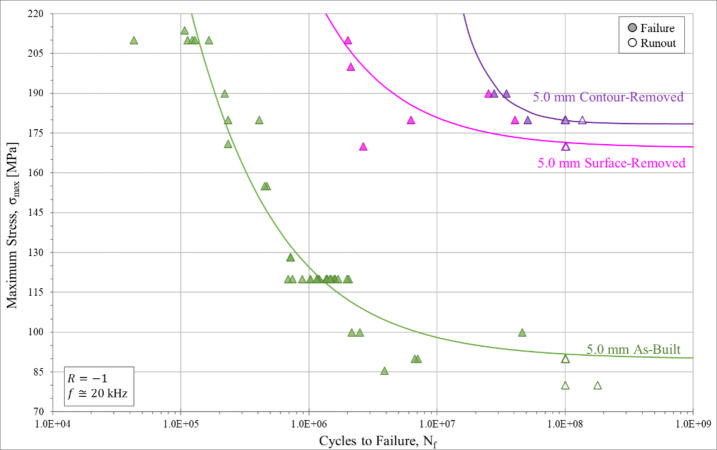
Stress-life (S-N) ultrasonic high cycle fatigue curves for surface-removed and contour-removed samples. Samples are shown compared to their 5.0 mm CL M2 as-built counterpart. Runout samples are indicated by unfilled data icons and are classified as cycling longer than 10^8^ cycles without failure. Each set of data is accompanied by a curve fit determined via random fatigue limit (RFL) analysis assuming a Weibull distribution.

The UF results for samples which had the as-built surface removed and the contour removed are shown in Fig. 8. Both the surface-removed and contour-removed results show a marked improvement in fatigue strength, S_N_, at 10^8^ cycles and fatigue life, N_f_ in all cycle regimes. The improvement is such that these 5.0 mm samples perform even better than the 2.5 mm and 1.5 mm as-built samples, demonstrating that the surface region has a substantial influence on the HCF behavior. This improvement is attributed to the removal of the surface crevices, as well as a general reduction in surface roughness. This surface removal reduces both the number of potential crack initiation sites and the severity of the stress concentration at these sites. A further slight improvement in fatigue strength was observed when the entire contour (including the contour/infill zone) is removed. In general, the fatigue strength estimates from the RFL analysis (Table 8) show no statistically significant difference between the surface-removed and contour-removed samples. It should be noted that the sample populations for both these groups is limited which makes estimating statistical significance of these small differences difficult.

**Table 8:** Fatigue strength for contour-removed and surface-removed sample groups compared to the as-built 5.0 mm diameter condition. Fatigue strength is determined by random fatigue limit (RFL) model and staircase testing procedure. The fatigue strength is defined as the stress needed to cause failure at 10^8^ cycles.

	Fatigue strength [MPa] via RFL	Fatigue strength [MPa] via staircase
5.0 mm contour-removed	179.0 *± 3.8*	181.4 *± 6.4*
5.0 mm surface-removed	170.0 *± 12.9*	175.0 *± 5.0*
5.0 mm As-built	89.5 *± 5.7*	91.0 *± 7.0*

**Fig. 9:**
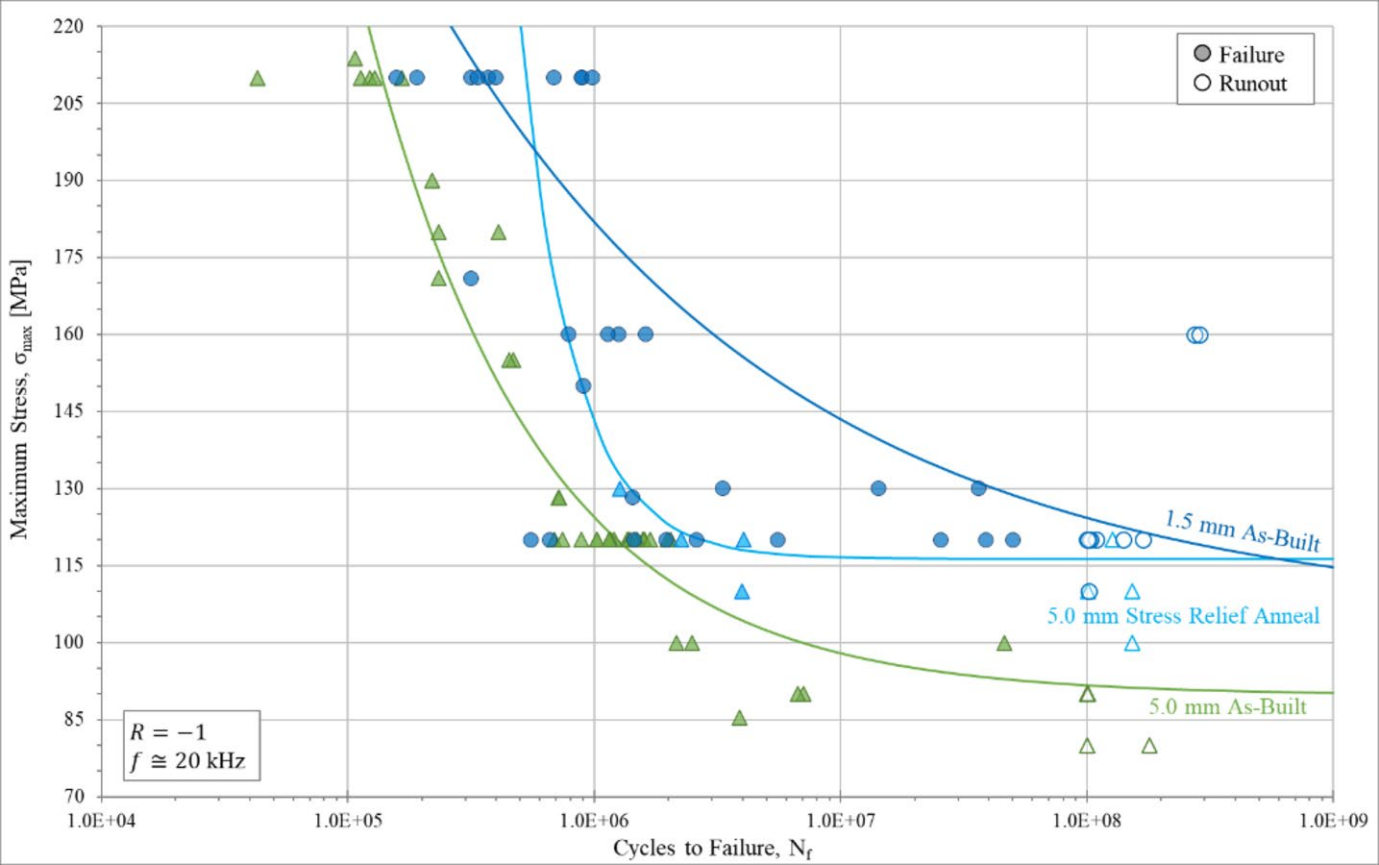
Stress-life (S-N) ultrasonic high cycle fatigue curves for as-built samples having undergone a stress relief anneal. All samples are shown compared to their 5.0 mm CL M2 as-built counterpart. Runout samples are indicated by unfilled data icons and are classified as cycling longer than 10^8^ cycles without failure. Each set of data is accompanied by a curve fit determined via random fatigue limit (RFL) analysis assuming a Weibull distribution.

The UF results for the stress relief annealed samples are shown in Fig. 9. The implementation of a stress relief anneal on as-built samples results in a moderate improvement in fatigue strength at 10^8^ cycles and a slight improvement in fatigue life. The stress relief anneal reduced the surface residual stress from 292 MPa to 143 MPa and improved the fatigue strength from 89.5 MPa to 116 MPa as shown in Table 9. For comparison, the surface residual stress in the as-built 1.5 mm samples is 69 MPa and the fatigue strength is 123 MPa.

.

**Table 9:** Fatigue strength for the stress relief annealed 5.0 mm sample group compared to the as-built 5.0 mm diameter and 1.5 mm diameter conditions. Fatigue strength is determined by random fatigue limit (RFL) model. The fatigue strength is defined as the stress needed to cause failure at 10^8^ cycles.

	Fatigue strength [MPa] via RFL	Fatigue strength [MPa] via staircase
1.5 mm As-built	123.0 *± 26.1*	122.5 *± 6.6*
5.0 mm Stress Relief Anneal	116.0 *± 4.9*	115.0 *± 8.7*
5.0 mm As-built	89.5 *± 5.7*	91.0 *± 7.0*

### Fracture surface analysis of as-built samples

**Fig. 10:**
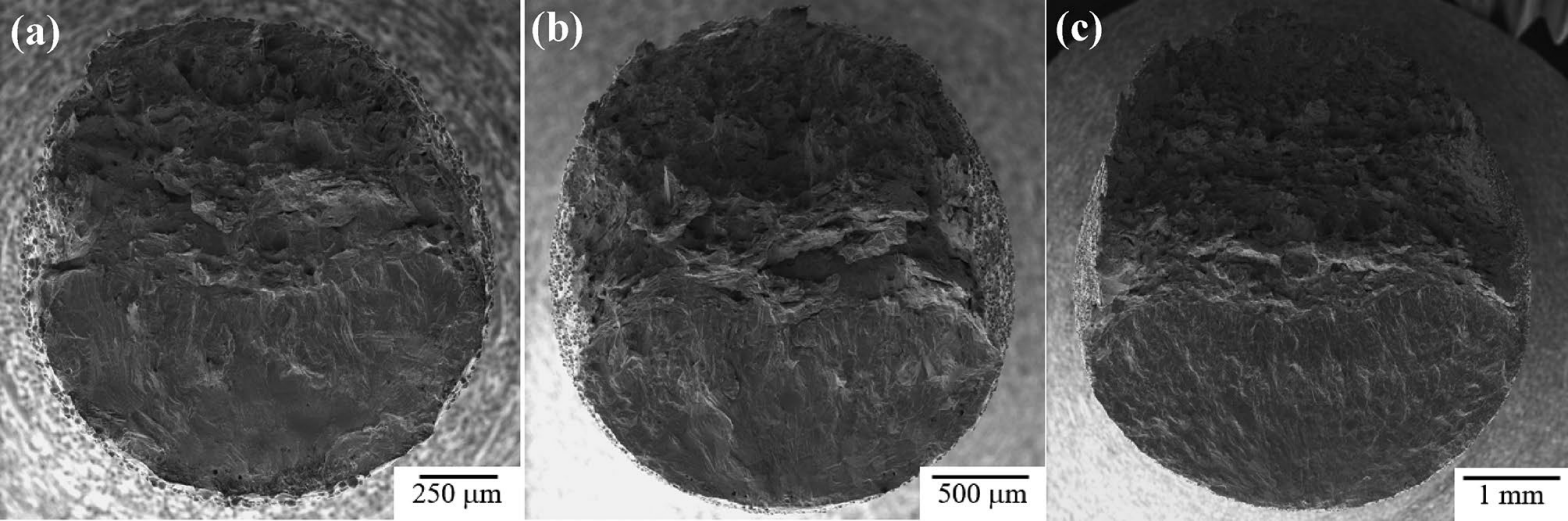
Representative ultrasonic fatigue fracture surface fractography for as-built (**a**) 1.5 mm CL M2, (**b**) 2.5 mm CL M2, and (**c**) 5.0 mm CL M2.

Fatigue fracture surfaces were characterized using SEM fractography. Figure 10 shows a representative view of the fracture surfaces for each as-built sample group. The fracture surface has two distinct regions: fatigue crack growth in the bottom region of the images and ductile overload in the top region of the images. The bottom regions show the path of crack growth caused by ultrasonic fatigue. The top region experiences ductile failure from manual overload by sample bending. This manual overload is done to reveal the entire fracture surface as the criteria for failure in the UF testing does not result in complete fracture of the specimen. As can be seen, the macroscopic fracture surface morphology is similar for all three gauge diameters.

**Fig. 11:**
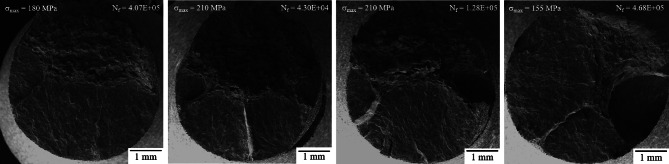
Multiple fatigue fracture surfaces found on 5.0 mm as-built high-stress, low-cycle samples.

While the images in Fig. 10 are the typical fatigue fracture surface seen in most samples, some of the 5.0 mm as-built samples showed multiple fatigue crack initiation sites as shown in Fig. 11. Evidence of multiple initiation sites were also seen in 2.5 mm and 1.5 mm samples, however much less common. In all cases, multiple fatigue fracture surfaces were most often seen in higher maximum stress, s_max_, conditions.

**Fig. 12:**
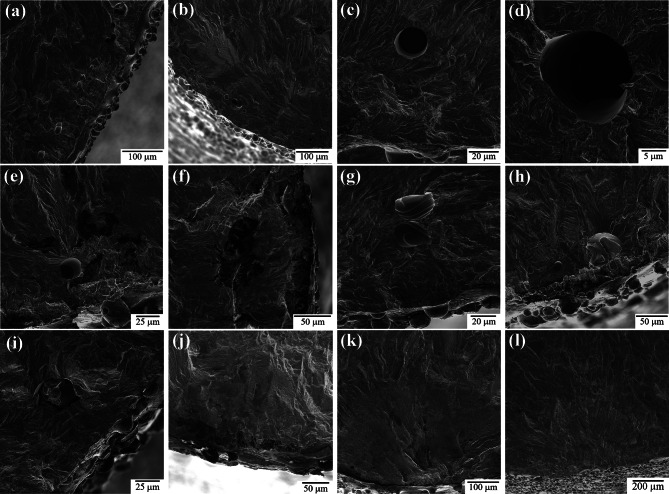
Examples of common defects seen on the fracture surface in each sample group. (**a**-**b**) increase in defect concentration at contour/infill region of CL M2 samples. (**c**) – (**d**) porosity via gas entrapment or keyholing found in CL M2 samples. (**e**) – (**f**) discontinuity in composition. (**g**) – (**h**) melt pool boundaries. (**i**) – (**j**) lack of fusion and irregular melting found in CL M2 samples. (**k**) – (**l**) surface initiation from surface crevice.

The fatigue fracture surface of each as-built sample reveals many different types of sub-surface defects, as highlighted in Figure 12. The primary defects were lack of fusion (LOF) porosity^27,48,84–87^, gas entrapment porosity^84,85,88,89^, keyhole porosity^25,90–94^, improperly melted particles^85,88,95–97^, and discontinuities in the composition^98^. For these samples, an increase in defect concentration was observed at the contour/infill region (Figure 12a & b), consistent with what was observed in EBSD (Figure 3) he defects in this region were a variety of improper melting causing both porosity (Figure 12c & d) and solid defects such as melt pool boundaries or unmelted particles (Figure 12g-i). Additionally, some of these samples have shown evidence of composition variation either due to a change in concentration of certain elements (typically increased carbon and decreased iron) or the inclusion of foreign elements (Figure 12e & f). Despite the abundance of these subsurface defects, the crack initiation in as-built samples were generally due to surface crevices or surface-connected defects (Figure 12k & l), not internal defects.

**Fig. 13:**
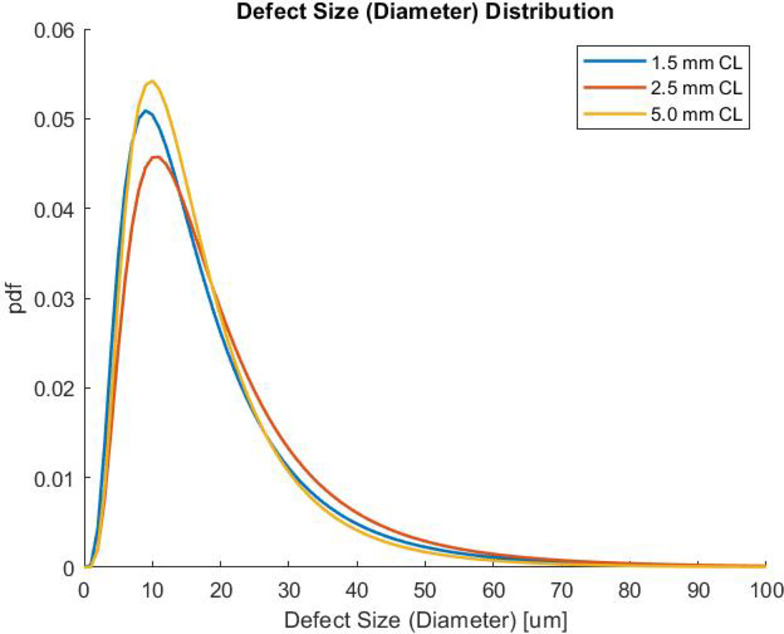
Defect size distribution is shown to compare the 1.5 mm, 2.5 mm, and 5.0 mm as-built samples. All defects on the fracture surface are measured. Defect size is quantified as the longest distance across the surface of a defect (diameter).

The size of the defects observed on the fracture surface were analyzed using ImageJ. Both the area and the longest distance across the defect (diameter) were measured. All defects were quantified in the same manner, regardless of whether they are a pore, inclusion, or irregular melting. In this part of the study, all defects in the as-built samples were measured regardless of whether or not they could be the initiating defect. This was done to determine if gauge diameter has an influence on the size of defects. It should be noted that in HCF of porous metals, it is generally accepted that the maximum pore size is the primary metric for fatigue life, rather than pore/defect density, since fatigue cracks will preferentially initiate at the largest defect/pore present^99–101^. Only obvious three-dimensional defects were characterized. Discontinuities associated with local composition differences (e.g., oxides, carbides, etc.) such as in Fig. 12f and near surface LOF such as in Fig. 12j have not been included in this population. This defect study also does not include or account for crack initiation at the surface due to surface defects, such as surface crevices or surface-connected defects (Fig. 12k & l), as the morphology of these defects cannot be seen from the fracture surface. The results for defect size (diameter), shown in Fig. 13, indicate that the size of defects is not affected by the gauge diameter. This effectively rules out processing defect size or defect morphology as being the leading cause of the gauge diameter effects on HCF observed on the as-built samples.

**Fig. 14:**
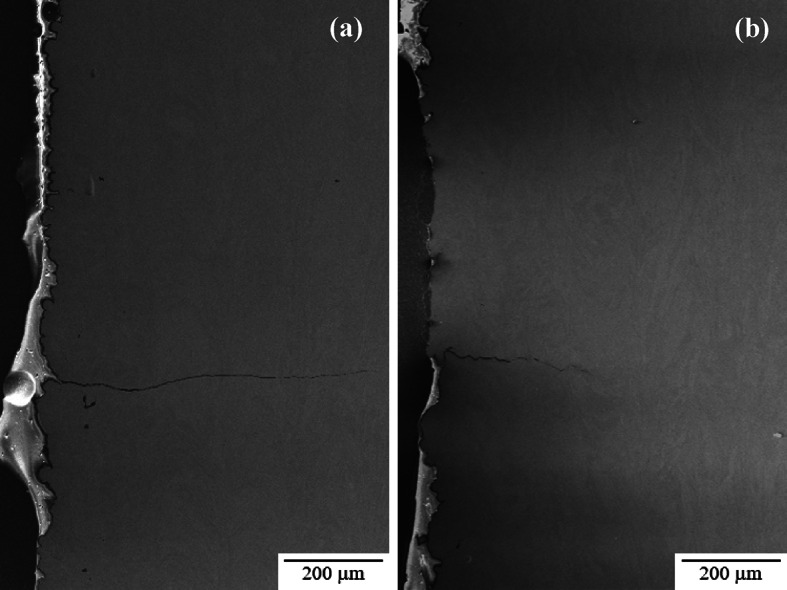
Example of crack initiating at surface crevice and propagating during UF testing in (**a**) 2.5 mm CL M2 and (**b**) 5.0 mm CL M2.

As stated previously, crack initiation in nearly every as-built sample occurs at or near the surface. Our investigation into surface roughness showed that there is no significant difference between gauge diameters, but that the presence of surface crevices must also be considered. The crevices are not detectable by the normal surface roughness measurements. The frequency of surface crevices in each sample set is difficult to determine as they are most readily observed in cross-sectioned samples which only isolates two locations around the circumference of the gauge section. The cross-section observations were inadequate to determine if the size or distribution of these crevices is affected by gauge diameter. However, it could be determined that in all as-built samples these crevices are the likely the source of crack initiation. Figure 14 shows examples of secondary cracks that had formed within the gauge section but away from the primary fatigue fracture surface in both 5.0 mm and 2.5 mm as-built samples. In both cases the cracks appear to have formed at a surface crevice.

**Fig. 15:**
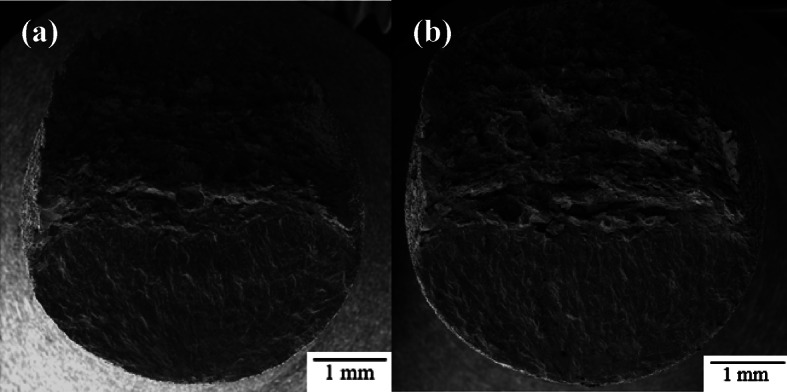
Representative ultrasonic fatigue fracture surface fractography for 5.0 mm CL M2 samples (**a**) as-built and (**b**) stress relief annealed.

Crack initiation also occurs at the surface region in the 5.0 mm stress relief annealed samples. Despite showing improvements in fatigue behavior due to the reduction of tensile stresses on the surface, the as-built surface finish remains the source of fatigue cracking just as in the as-built samples. For this reason, the fracture surfaces of both sample groups are largely indistinguishable, as shown in Fig. 15.

### Fracture surface analysis of samples with as-built surface regions removed

**Fig. 16:**
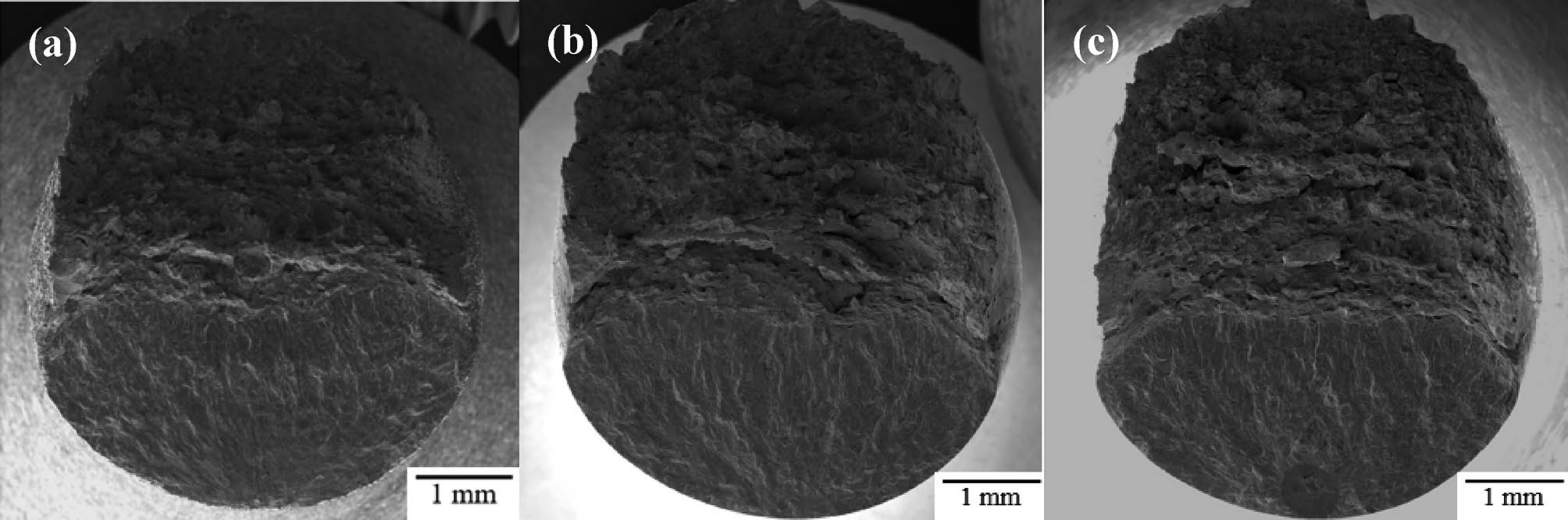
Representative ultrasonic fatigue fracture surface fractography for 5.0 mm CL M2 samples (**a**) as-built, (**b**) surface removal, and (**c**) contour removal samples.

To summarize what is known so far: gauge diameter effects on fatigue behavior have been shown to occur in 316 L L-PBF. It is also shown that the microstructure, surface roughness, defect morphology, and defect size distribution do not change with changing gauge diameter. In addition to this, it is shown that a concentration of defects as well as a change in microstructure occurs at the contour/infill zone but that crack initiation generally occurs at or near the surface. For this reason, an investigation was conducted to analyze the influence the as-built surface morphology and the contour/infill zone morphology has on the fatigue behavior. Figure 16 shows a representative fracture surface of 5.0 mm surface-removed and contour-removed samples, compared to the as-built fracture surface. The general shape of the fracture surface is nominally the same as the as-built samples, but the source of crack initiation is starkly different.

**Fig. 17:**
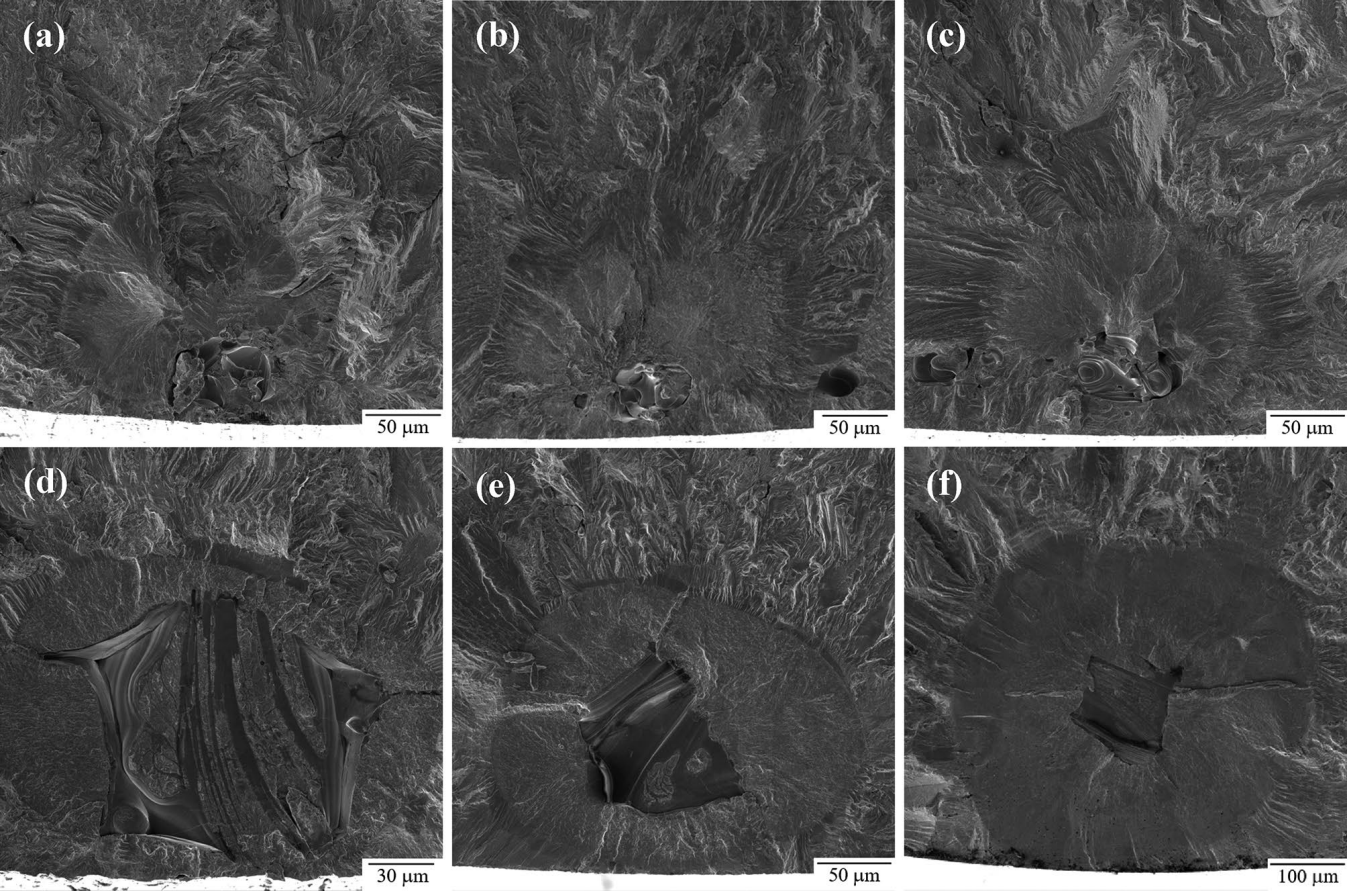
Examples of common initiating defects seen in (**a**-**c**) surface removal and (**d**-**f**) contour removal samples.

While the as-built samples typically showed crack initiation occurring at or near the surface, the surface- and contour-removed samples show sub-surface, internal defects as the source of crack initiation. Figure 17 shows the general initiation site morphology of both surface- and contour-removed samples. The initiation sites in the surface-removed samples were generally from AM processing defects that can be characterized as a complex conglomerate of lack of fusion (LOF) and melt pool defects. Evidence of these types of defects were also seen on the fracture surface of as-built samples, however they were rarely the source of crack initiation. The initiation site in the contour-removed samples has a defect morphology not reported by previous researchers. There is a distinct "fish-eye" surrounding each defect, showing that initial crack growth occurred sub-surface and in vacuum^4,102^. Once the crack reaches the surface, the fracture surface morphology changes to that of a typical "in-air" crack path. These defects tend to be larger and further from the surface than the defects found in the surface-removed samples. They also all tend to have a roughly square shape and a relatively flat surface. The morphology of the defect surfaces suggest that these are LOF voids between melt pool boundaries, however the reason for this shape is unknown

**Fig. 18:**
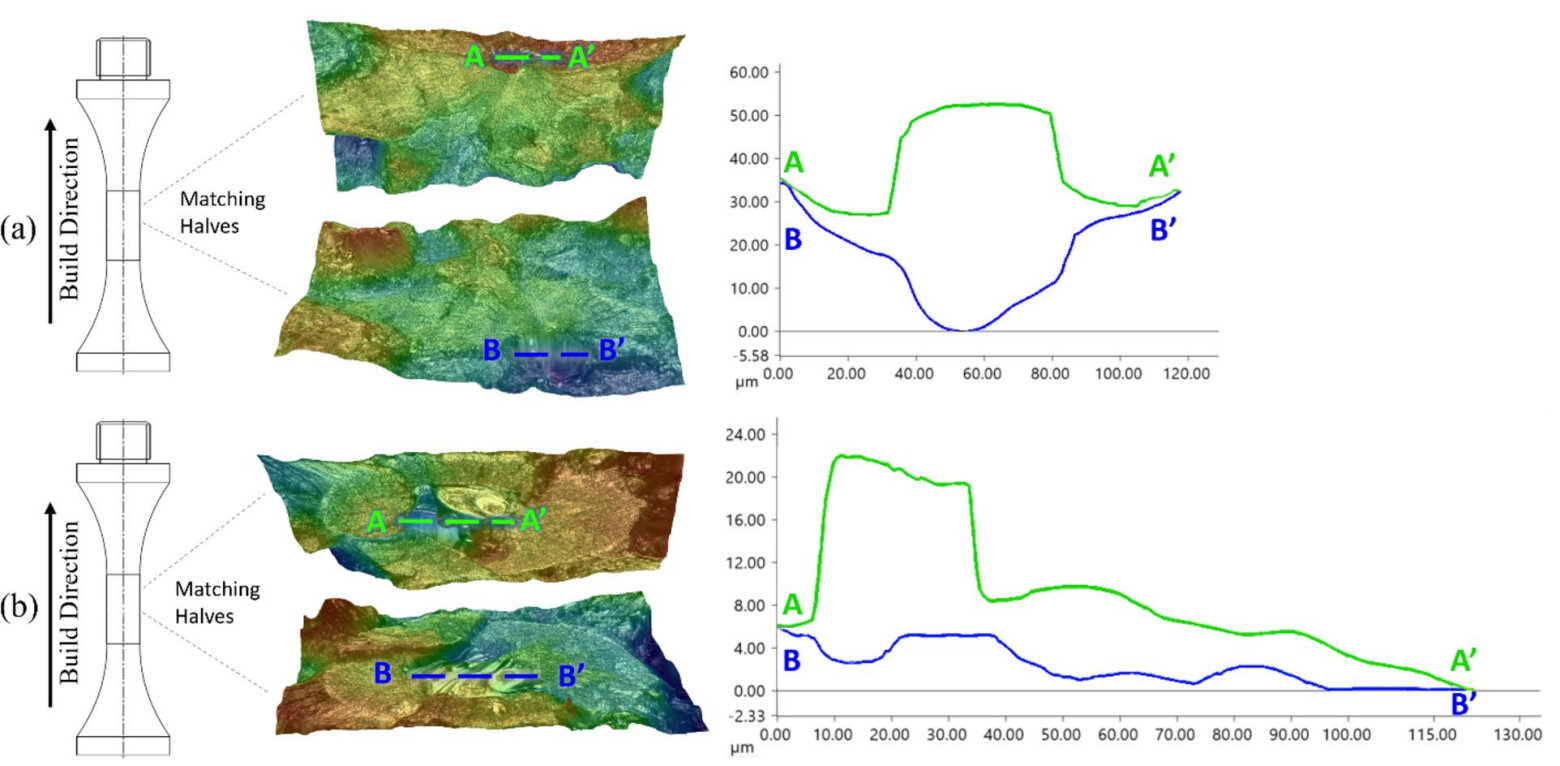
3D topological maps of both matching halves for a fractured (**a**) surface-removed sample and (**b**) contour-removed sample. A cross-sectional profile is taken for each sample at the location indicated by the dashed lines and is plotted to depict the total height of a LOF defect. The topological map and line profiles are generated using the Keyence microscope and its associated software as referenced in Sect. 2.5.

The initiating defects for the surface-removed and contour-removed samples can be further compared by looking at the 3D morphology of the LOF defects. Fracture surface topology and profilometry were conducted on all failed samples using the Keyence optical microscope to assess the defect height in the build direction. Matching halves of a given fractured sample are shown in Fig. 18 to help visualize the 3D morphology of the initiating defect. The average height (along the build direction) for an initiating defect is 46 μm for a surface-removed sample and 19 μm for a contour-removed sample. The cross-sectional area of the initiating defect is on average 3,803 µm^2^ and 11,335 µm^2^ for surface- and contour-removed samples, respectively. While the initiating defect in both sample groups were determined to be LOF defects, the initiating defects in the contour-removed samples were more expansive across a given build layer but rarely permeates through multiple build layers – a build layer being 25 μm thick. The defects found in the surface-removed samples almost always extended through multiple build layers, suggesting a different formation mechanism.

**Fig. 19:**
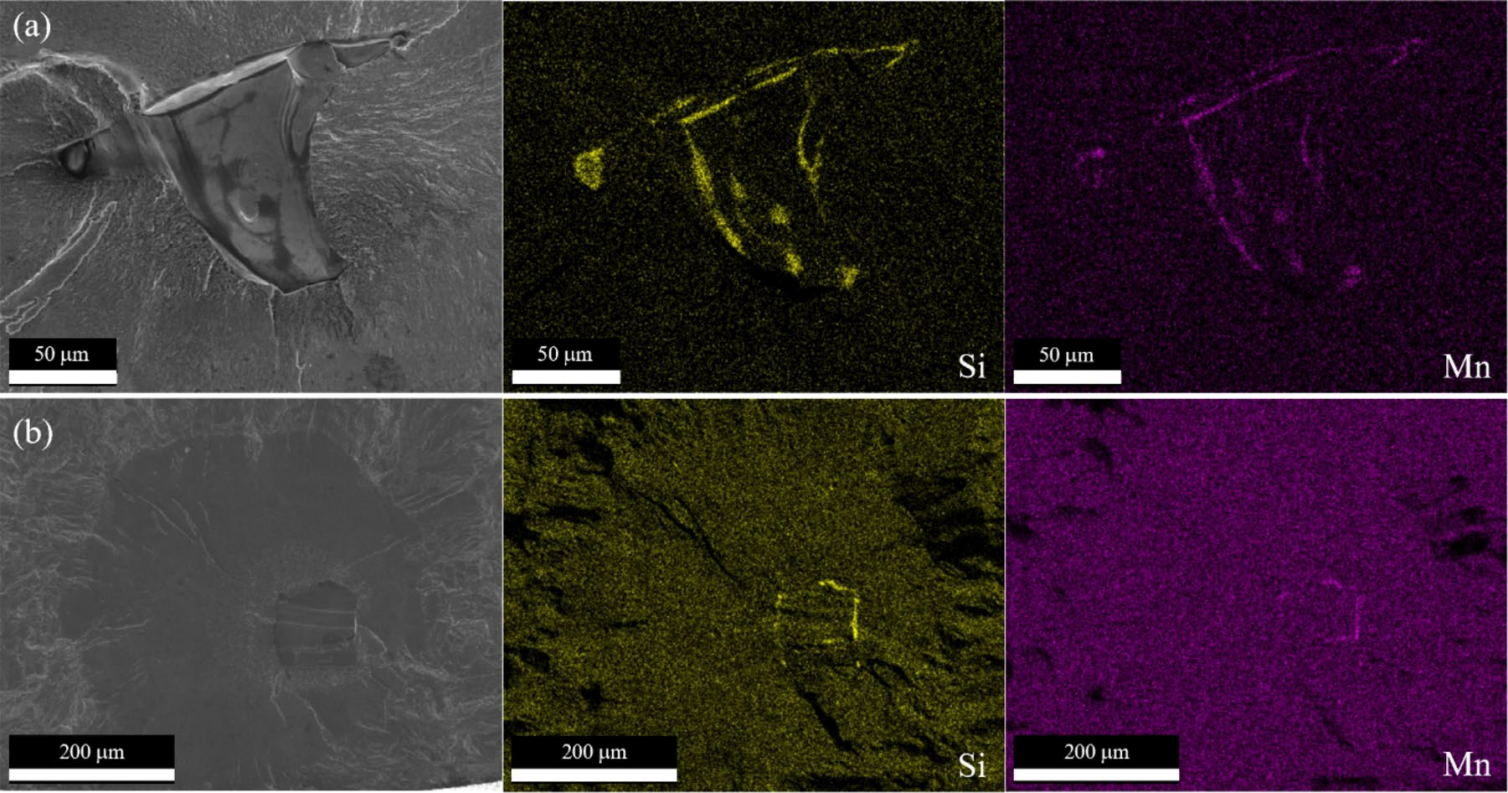
LOF structure of initiating defects in contour removal samples. (**a**) and (**b**) SEM micrographs of initiating defects with their corresponding Si and Mn EDS elemental maps.

Since the LOF defects in surface-removed and contour-removed samples differed morphologically, composition analysis was conducted to provide insight into the defect formation mechanisms. Composition analysis via EDS for the surface-removed initiating defects shows an essentially uniform composition across the initiating defect and fracture surface. The contour-removed samples, on the other hand, show compositional variation specifically at the LOF defects (Fig. 19). EDS shows increased concentrations of Si and Mn, indicative of Si-oxides (SiO_2_), Mn-oxides (MnO), and silicates (MnSiO_3_, Mn_2_SiO_4_)^103,104^. This suggests that these silicate-oxides form due to Si and Mn reacting with O trapped in the powder particles and/or O trapped in the voids of LOF defects.

**Fig. 20:**
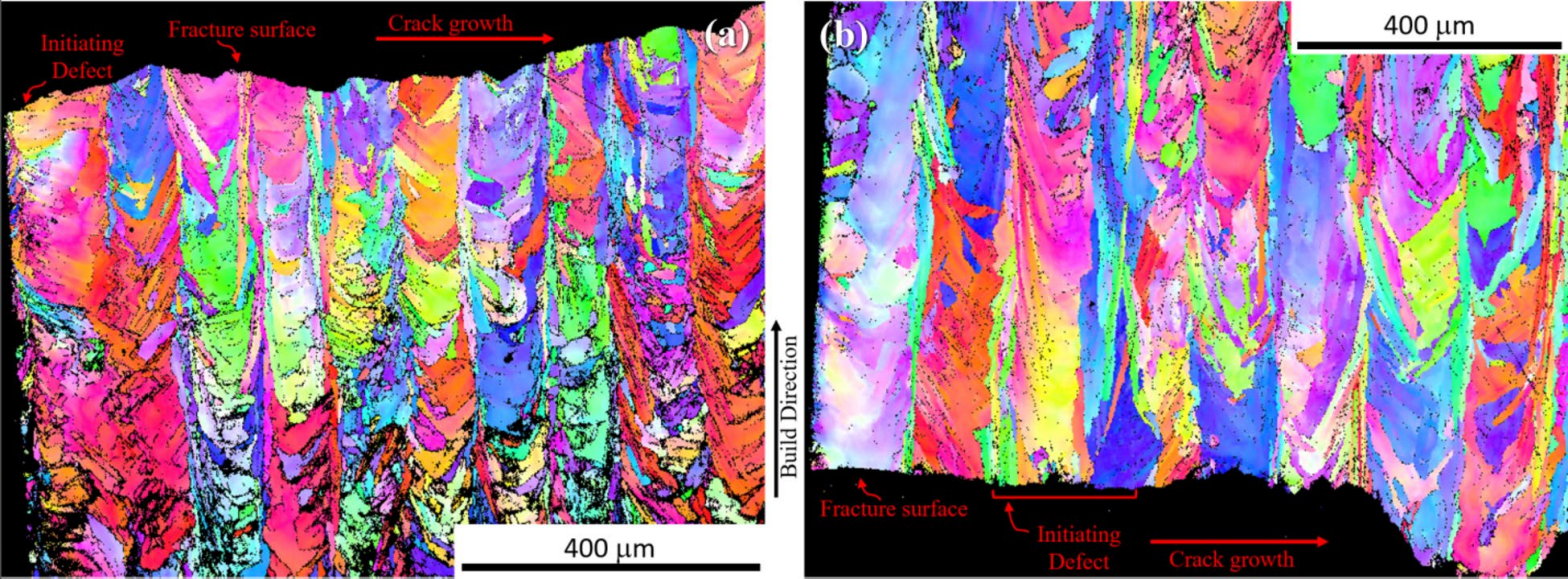
EBSD inverse pole figure (IPF) showing the microstructure beneath the initiating defect and fracture surface for (**a**) surface removal sample, and (**b**) contour removal sample. The left side of each image shows the sample surface. The surface removal sample shows the contour/infill zone still intact while the contour removal sample shows no evidence of the contour. Figures were generated using EDAX OIM Analysis as referenced in Sect. 2.8.

To further investigate 20the differences between the surface-removed and contour-removed samples, the microstructures beneath the fracture surfaces are compared via EBSD. Figure depicts a cross-sectional view of a surface-removed sample (Fig. 20a) and contour-removed sample (Fig. 20b). These images show both the external ground surface and the path of crack growth from the initiating defect. Figure 20a shows that part of the contour and the contour/infill zone remain intact in the surface-removed sample, while Fig. 20b shows that in the contour-removed sample this region has been removed. The surface-removed sample shows a crack growth path that is relatively tortuous, as opposed to the contour-removed sample which has a smooth crack growth path. This suggests different crack growth mechanisms and behavior between the two sample types, which would help explain why the contour-removed specimens have longer fatigue lives despite having larger initiating defects.

## Discussion

In this study we have characterized the influence of AM section thickness on HCF, using fatigue sample gauge diameter as a measure of section thickness. A pronounced gauge diameter effect on HCF in AM 316 L stainless steel has been observed. In particular, 1.5 mm diameter samples show a substantial increase in fatigue life and fatigue strength at 10^8^ cycles compared to 5.0 mm samples. The HCF response of 2.5 mm diameter samples was intermediate to these results. We have also attempted to identify the underlying factors that produce this gauge diameter effect as discussed below.

**Fig. 21:**
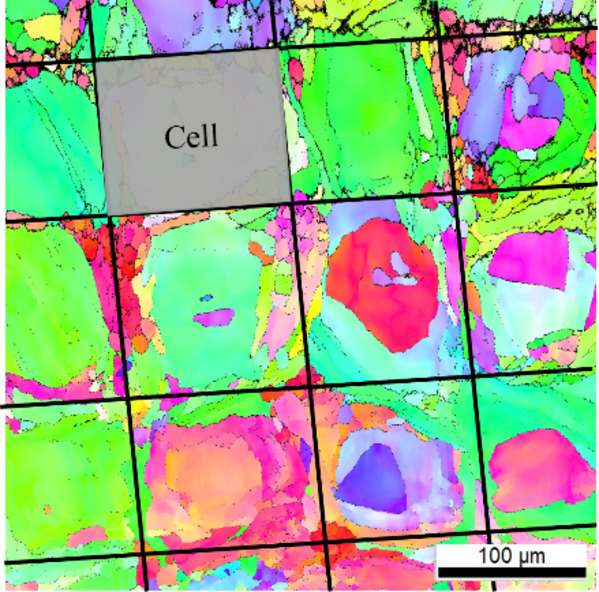
Depiction of what constitutes a cell in the microstructural analysis. The size of the cell (width) is used to qualitatively represent microstructural changes in each build.

Previous studies [e.g., 14,16] have shown that adjusting the processing parameters can alter the microstructure, however, in the current investigation, the processing parameters were not altered for the different gauge diameter fabrication, therefore changes in microstructure with gauge diameter were not expected and were not observed. Additionally, gauge diameter did not have an observable effect on the microstructure as shown in Fig. 3. Normal to the build direction, all groups have a crosshatch pattern (Fig. 3e-f, i-j), characteristic of directional scanning strategies in L-PBF. Parallel to the build direction, columnar grains that extend across multiple build layers were observed. This cross-hatched cell microstructure is common in many L-PBF systems as grains will preferentially grow in the direction of heat flow (along the build direction) and correlate with melt pools which span multiple layers. A cell represents a given grain or grain cluster that matches with a single square in the crosshatch pattern, as demonstrated in Fig. 21. Comparing cell sizes for each gauge diameter (Fig. 3b & f), it can be observed that the cells are roughly the same size at approximately 125 μm in width in the infill regions. Additionally, the melt pool depth (Fig. 3d & h) and width of the contour zone (Fig. 3a, c, e, g) appears unchanged between the 1.5 mm and 5.0 mm as-built samples. If only microstructure was taken into consideration, one may not expect any change in fatigue with changing gauge diameter. Since we do see changes in fatigue behavior with gauge diameter, this indicates that factors other than the mesoscale microstructure dominate the HCF response of these samples.

Processing-related AM defects found on the entire fracture surface were characterized by SEM and determined to be consistent with lack-of-fusion porosity and solid defects such as melt pool boundaries or unmelted particles. These defects were not associated with fatigue crack initiation but were taken to be indicative of the general porosity from AM. Quantification of these images indicated that the size of these defects follows a log normal distribution and both the size and distribution are statistically indistinguishable for all three gauge diameters. The mean size of these defects was approximately 34 μm in diameter. In addition, an increased concentration of defects was observed at the infill/contour zone for all sample diameters. This suggests that defect sizes, distribution, and morphology do not significantly impact the gauge diameter effect on fatigue behavior of these as-built samples.

For samples tested in the as-built condition, crack initiation is observed to occur at the as-built surface in the majority of cases. These cracks generally were related to surface connected crevices that are assumed to have formed during individual AM processing passes and can be considered AM processing-related defects. Fatigue cracks generally initiate at areas of highest stress concentration. The geometry and location of the surface crevices in the as-built specimens is such that crack initiation is likely to occur at these sites. There did not appear to be a correlation between crevice depth and sample gauge diameter, so this did not appear to explain the gauge diameter influence on fatigue lives. The measured value of the as-built surface roughness showed no significant difference between gauge diameters, indicating that surface roughness does not significantly impact the gauge diameter effect on fatigue behavior.

While the processing parameters themselves were unchanged for the different builds, the complexity of the thermal history required to produce different gauge diameters can affect the residual stresses produced in each build set. All as-built samples, regardless of gauge diameter, demonstrate a peak tensile axial residual stress near the surface and transition into a peak compressive axial residual stress at the sample interior. This type of residual stress distribution has been observed previously for L-PBF processes^47,51–54^. The difference between gauge sizes lies in the magnitude of residual stresses, which shows a higher magnitude near-surface tensile stress in the 5.0 mm as-built samples and a higher magnitude sub-surface compressive stress in the 1.5 mm as-built samples. Tensile residual stresses have been shown to negatively impact fatigue life by increasing the local mean stress and assisting in crack opening^46^. Our results are consistent with at least one previous study^47^. Wu et al. reported that a reduction in part thickness can result in a lower magnitude tensile axial residual stress on the surface^47^. It should be noted that they also reported a lower magnitude compressive stress in the thinner region of the sample interior. Our observation is the opposite of this with a somewhat lower magnitude of compressive residual stresses in the sample interior for the 1.5 mm sample compared with the 5.0 mm sample. To ensure resonance of the ultrasonic fatigue samples, changes in sample diameter also require changes in the length of the sample scaled with the gauge diameter. Mercelis and Kruth found that the more layers added (more layers results in a taller height) the higher the residual stress is in the final part^53^. Our results are consistent with this observation, in that the longer gauge length in 5.0 mm samples correlated with a higher tensile residual stress on the surface.

The residual stress analysis in this current work showed that the axial residual stress is of greater magnitude and is tensile on and near the surface for 5.0 mm samples compared to 1.5 mm samples which exhibited a lower tensile residual stress on the surface. This could contribute to the observed gauge diameter effect, but it is important to note the degree to which the tensile residual stress negatively affects the fatigue behavior. Fatigue testing shows a moderate improvement in fatigue strength at 10^8^ cycles and slight improvement in fatigue life with a stress relief anneal of the 5.0 mm as-built samples (Fig. 9b). Additionally, fatigue crack initiation occurs at or near the surface, in the same manner as the as-built samples. Since the crack initiation sites and general fracture surface appear to be unaffected by gauge diameter, the improvement in fatigue behavior is attributed to the reduction in tensile residual stresses at the sample surface in the 1.5 mm samples. The higher magnitude tensile residual stress observed in the 5.0 mm sample would in general increase the local mean stress and/or crack opening stress during fatigue testing resulting in shorter fatigue life. The strong correlation between residual stress distribution and fatigue behavior is consistent with previous work. Lueders et al.^30,33^ has shown that the tensile residual stresses present in L-PBF parts negatively impact the fatigue behavior in the high cycle regime as it affects crack growth. Subasic et al.^41^ has shown that reducing the surface tensile residual stresses by means of tensile preloading results in an increased fatigue limit for 316 L specimens.

In the current study, experiments conducted in samples subjected to a stress relief heat treatment showed a pronounced reduction in the residual stresses and also improvements in the HCF behavior. This further demonstrates the importance of residual stresses on fatigue behavior. This observation is consistent with previous studies^32,79,80,82^.

For fatigue critical AM components, the results of this study show the importance of controlling residual stresses that are produced during AM fabrication. Without mitigating residual stress formation, AM components of varying section thickness can be expected to have significant variations in HCF lives, with thick sections exhibiting an increase in the probability of fatigue failure. The effect of section thickness can be somewhat moderated by subjecting AM components to stress relief heat treatment.

## Conclusions

In this study, the fatigue behavior of L-PBF 316 L SS under fully reversed ($\:R=-1$) ultrasonic fatigue loading in the high to very high cycle fatigue regime was investigated. Cylindrical dogbones of three gauge diameters (1.5 mm, 2.5 mm, 5.0 mm) were fabricated in the vertical direction with no post-processing to study the effects of sample diameter on fatigue behavior in the as-built condition. An additional set of samples were fabricated for post-processing for the purpose of studying the effects of the surface removal, contour removal, and stress-relief anneal on HCF in the 5.0 mm diameter samples. The following conclusions can be drawn:

1. The high to very high cycle ultrasonic fatigue behavior of these AM samples is strongly influenced by the diameter of the specimen. A reduction in gauge diameter results in an increased fatigue strength at 10^8^ cycles and increased fatigue lives in the high cycle fatigue regime.
2. For samples built on the same AM machine (Concept Laser M2) and using the same processing variables, the fabrication of samples with varying diameter does not result in differences in microstructure (grain size, morphology and texture), defect size and distributions, or surface roughness. This indicates that the mesoscale microstructure and defect structure are not responsible for the observed changes in fatigue behavior.
3. Fractography suggests that crack initiation occurs at or near the surface in as-built samples. Cross-section analysis revealed that the surface initiation is likely due to deep surface crevices.
4. Axial residual stress magnitudes are affected by the gauge diameter, and likely contribute to sample diameter effect. Large diameter samples have a higher magnitude tensile residual stress near the surface, while smaller diameter samples have a higher magnitude compressive residual stress at the center of the samples. Partial relaxation of residual stress via stress relief anneal demonstrates that the high magnitude tensile stresses on the surface negatively affects the high cycle fatigue behavior.
5. Removing the as-built surface significantly improves the fatigue life and fatigue strength at 10^8^ cycles. The fatigue behavior is further improved when the contour/infill zone is removed. In samples which have had the surface removed, fatigue crack initiation occurs at relatively large lack-of-fusion defects that are typical of AM processing.

## Data Availability

The experimental data are available on Materials Commons https://materialscommons.org/ and can be found at the 10.13011/m3-c93d-z035.
